# No-Reference Objective Video Quality Measure for Frame Freezing Degradation

**DOI:** 10.3390/s19214655

**Published:** 2019-10-26

**Authors:** Emil Dumic, Anamaria Bjelopera

**Affiliations:** 1 Department of Electrical Engineering, University North, 42000 Varaždin, Croatia; 2 Department of Electrical Engineering and Computing, University of Dubrovnik, 20000 Dubrovnik, Croatia; anamaria.bjelopera@unidu.hr

**Keywords:** NR-FFM, frame freezing degradations, no-reference quality measure, RVQM, STRRED, VQM-VFD

## Abstract

In this paper we present a novel no-reference video quality measure, NR-FFM (no-reference frame–freezing measure), designed to estimate quality degradations caused by frame freezing of streamed video. The performance of the measure was evaluated using 40 degraded video sequences from the laboratory for image and video engineering (LIVE) mobile database. Proposed quality measure can be used in different scenarios such as mobile video transmission by itself or in combination with other quality measures. These two types of applications were presented and studied together with considerations on relevant normalization issues. The results showed promising correlation values between the user assigned quality and the estimated quality scores.

## 1. Introduction

Quality of media content can be evaluated using different types of measures. The most reliable way of doing this is by conducting visual experiments under controlled conditions, in which human observers grade the quality of the multimedia contents under evaluation [1]. Unfortunately, such experiments are time-consuming and costly, making the search for alternative quality estimation methods an important research topic. A much simpler approach is to use some computable objective measure that equates quality degradation with the (numerical) error between the original and the distorted media [2,3]. Every objective quality measure has as its aim approximating the human quality perception (or human visual system, HVS) as closely as possible, meaning that good correlation with subjective measures (mean opinion score, MOS) is sought. Objective quality measures for image and video can be generally divided into three categories according to the reference information they use, as follows:

- full-reference (FR) quality measures;
- reduced-reference (RR) quality measures;
- no-reference (NR) quality measures.

FR quality measures require the original undistorted or unprocessed signal. NR quality measures require only the processed/degraded signal. RR quality measures need information derived from the original signal [3].

In this paper, we present no-reference video quality measure for frame freezing degradations called NR-FFM (no-reference frame–freezing measure). This type of degradation often occurs during video transmission in different types of TV broadcasting (internet, mobile), with low SNR (signal to noise ratio) margin. It will be shown that the proposed measure achieves high correlation with MOS and that it can be used in cases where only this type of degradation is expected or in combination with other degradation types, so that NR-FFM is combined with some other FR or RR objective measure.

This paper is organized as follows: Section 2 describes related work, Section 3 describes the test of the laboratory for image and video engineering (LIVE) mobile database, Section 4 describes the development of the proposed NR-FFM measure, Section 5 presents the experimental results, Section 6 provides the discussion, and Section 7 draws the conclusions.

## 2. Related Work

A survey that classifies and compares current objective video quality assessment algorithms can be found in [4]. Generally, any image quality measure can be extended to video sequence analysis by computing the quality difference of each image of the distorted video to its corresponding image in the original video (or by computing the quality of each image of the distorted video alone, for NR measures). These measures include the well-known PSNR (peak signal-to-noise ratio) [5], SSIM (structural similarity index) [6], VSNR (visual signal to noise ratio) [7], and reduced reference image quality assessment meter (IQAM) [8], among others. Because these image-based measures are not able to capture temporal distortions, different full reference or reduced reference video quality measures for simultaneous capturing of spatial and temporal distortions have been developed. These measures include MOVIE (motion-based video integrity evaluation) [9], VQM (video quality measure) [10], STRRED (spatio-temporal reduced reference entropic differences) [11], and RVQM (reduced video quality measure) [12], among others. VQM accounting for variable frame delay distortions (VQM-VFD) have been also proposed in [13].

Because frame-freezes produce video sequences that may have a total play time longer than reference video sequence (e.g., in the case of stored video delivery), it is unclear how to compare such video sequences by using full or reduced reference quality measures. Also, by matching segments of the degraded sequence to corresponding segments of the reference, usual quality measures would always be perfect (because the sequences tested, from the LIVE mobile database, have no other degradations besides frame freezing).

The paper [14] presents a subjective test that estimates the quality of video subject to frame freeze and frame skip distortions, showing that they impact the video quality differently. The paper [15] describes a no-reference temporal quality measure to model the effect of frame freezing impairments on perceived video quality; however, their subjective tests results are not available, preventing comparison with our proposed measure NR-FFM. In the paper [16], a new objective video quality measure based on spatiotemporal distortion is described. However, this measure also takes into account other degradations besides frame freezes and so the quality estimate reflects the combination of these effects. For sequences with frame freezing degradation only, this measure would calculate the same objective grade (even with different AFR (affected frame rate) for frame freezing degradations). The paper [17] tested video quality model (VQM) that accounts for the perceptual impact of variable frame delays (VFD) in videos. VQM-VFD demonstrated top performance on the laboratory for image and video engineering (LIVE) mobile video quality assessment (VQA) database [18].

There are also other algorithms for the detection of dropped video frames. In one of the state-of-the-art algorithms [19], the author proposed a jerkiness measure that is also a NR method. Jerkiness is a perceptual degradation that includes both jitter (repeating of frames and dropping frames) and freezing (exceedingly long display of frames). The jerkiness measure takes two variables into consideration: the display time and the motion intensity. It is a sum of the product of relative display time and a monotone function of display time, as well as a monotone function of motion intensity over all frame times. This measure is also applicable to a variety of video resolutions. In [20], the motion energy of videos is used in order to detect dropped video frames. The algorithm calculates temporal perceptual information (TI) and compares it to a dynamic threshold in order to detect long and short duration of frame repetition. However, the problem with this NR algorithm is the periods of low motion causing false-positive results, which is the reason why the RR version is better to apply. The authors in [21] use algorithms from [20] and [15] for comparison and in terms per frame computation times, their algorithm outperforms the mentioned NR methods. This algorithm uses two separate thresholds for videos with high and low motion content. The NR algorithm in [22], aside from [20] and [15], uses the algorithm in [19] for comparison. However, the comparison with [15] did not show good results in cases of freeze duration less than 0.5 s, and thus the focus was put on [20] and [19]. This algorithm [22] showed better performance in terms of estimating the impact of multiple frame freezing impairments and is closer to subjective test results. The new NR method in [23] that measures the impact of frame freezing due to packet loss and delay in video streaming network is based on [21] with a few modifications. This method was also tested on three more databases and detailed comparison is given for the algorithm in [20]. The study in [24] showed that the algorithm in [20] has better performance. A recent work from [25] is a novel temporal jerkiness quality metric that uses the algorithm from [20] and neural network for mapping between features (number of freezes, freeze duration statistics, inter-freeze distance statistics, frame difference before and after the freeze, normal frame difference, and the ratio of them) and subjective test scores. In recent papers [26,27], algorithms were tested on UHD (ultra high definition) videos. In [26], the histogram-based freezing artifacts detection algorithm (HBFDA) is proposed. HBFDA methodology includes comparison of consecutive video frames which consist of splitting a frame into regions and then comparing the regions’ consecutive frame histograms. The HBFDA algorithm adapts its parameters in real-time and can process 75 frames per second. The authors of [27] propose a new real-time no-reference freezing detection algorithm, called the RTFDA. A detailed comparison of results is given for the algorithm in [20]. RTFDA has a high detection rate of video freezing artefacts (in videos of different content type, resolution, and compression norm) with low false-positive rate in comparison to other commonly used freezing detection algorithms. It achieves real-time performance on 4K UHD videos by processing 216 frames per second.

In this study, we developed a no-reference measure for frame-freezing degradations (NR-FFM) on the basis of AFR and spatial information of the video content. NR-FFM can be used to quantify frame freezing degradations alone, or in combination with other measures to calculate quality in the presence of different types of degradations. LIVE mobile database [18] was used for NR-FFM evaluation. Additionally, the VQEG (Video Quality Experts Group) HD5 dataset [28] was also used to further test NR-FFM measures using a new set of video sequences.

Compared to the existing measures, contributions in this paper are:

- NR-FFM measure based on overall frame duration, number of frame freezings, and spatial information of the tested video sequence;
- NR-FFM obtains best correlation among other tested frame freezing objective measures in LIVE mobile dataset and similar correlation in VQEG HD5 dataset as other tested measures;
- NR-FFM can be combined with other objective measures designed for other degradation types, tested on LIVE mobile dataset with five degradation types, and combined with RVQM and STRRED as reduced reference measures.

## 3. Description of Used Datasets with Subjective Ratings

### 3.1. LIVE Mobile Dataset

To develop and test the proposed measure NR-FFM, we used sequences from the LIVE Mobile Video Quality Database [18] (later called LIVE mobile) using data from the mobile study and from the tablet study. The 10 video files in mobile study (out of which the first 5 were used in tablet study) are stored in planar YUV 4:2:0 format, with a spatial resolution of 1280 × 720 pixels and 30 fps and each is 15 s long. There are 20 degraded video sequences per original sequence with 5 distortion types (altogether 200 distorted video sequences in the mobile study and 100 video sequences in the tablet study):

- H.264 compression (four test videos per reference);
- Wireless channel packet-loss (four test videos per reference);
- Frame-freezes (four test videos per reference);
- Rate adaptation (three test videos per reference);
- Temporal dynamics (five test videos per reference).

**Figure 1:**
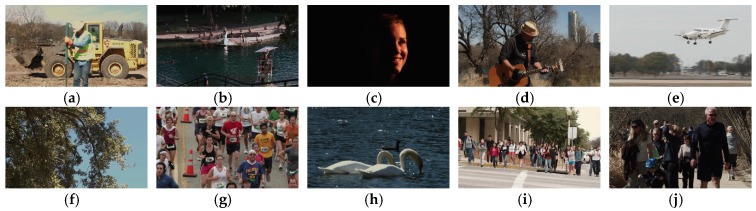
First frame, laboratory for image and video engineering (LIVE) mobile database: (**a**) bulldozer with fence, (**b**) Barton Springs pool diving, (**c**) friend drinking Coke, (**d**) Harmonicat, (**e**) landing airplane, (**f**) panning under oak, (**g**) Runners skinny guy, (**h**) two swans dunking, (**i**) students looming across street, (**j**) trail pink kid.

The first frame from each video sequence is shown in Figure 1.

**Figure 2:**
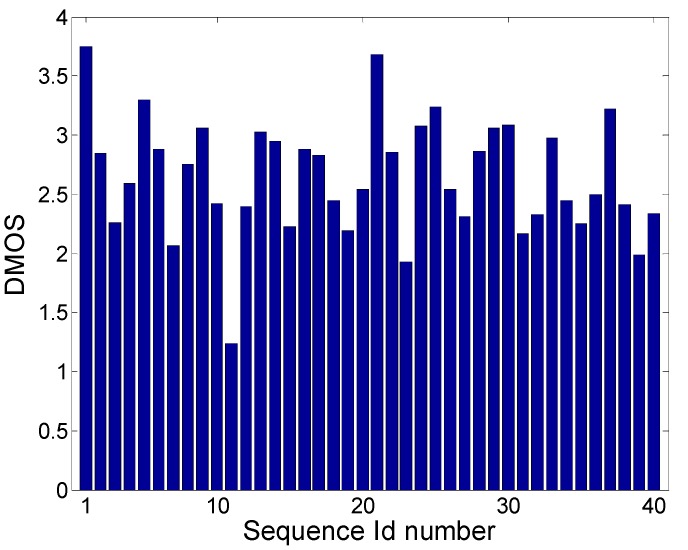
DMOS scores on LIVE mobile database frames with freezing degradation only.

In [18], the authors compared different objective measures using four degradation types from the dataset, omitting frame freezing degradation. In our study, we developed a no-reference objective measure for frame-freezing degradations, which was tested using 10 degraded sequences and 4 degradation levels, obtained from LIVE mobile database [18] (mobile subjective testing)—the first 3 degradations simulated the transmission of stored video material, which meant that after a freeze, playing re-started from the next frame in time, whereas the fourth degradation simulated live streaming, for example, after a frame freeze, all skipped frames were lost. Affected frame rate (AFR) was the same for the first three degradations, for example, the first degradation had 8 × 30 freezing frames, the second degradation had 4 × 60, and the third had 2 × 120 freezing frames; AFR was 240/690 = 34.78%. The fourth degradation had 1 freezing episode in the middle of the sequence lasting 120 frames, and 1 frame freeze at the end of the sequence lasting 60 frames; AFR was 180/450 = 40%. The first 3 s of all degraded video sequences were without freezing and duration of non-freezing video parts, set to be 1.5× the duration of the freezing parts. Figure 2 shows a graph with the DMOS (differential mean opinion score) results for these sequences. All 40 degraded videos had only frame freezing degradations, with no other degradation types included in them.

Several conclusions can be drawn from the graph (similar conclusions can be found in [14] where authors used a different database):

- DMOS score was lower (better) for longer frame freezing in the case of the stored streaming scenario (degradation types 1–3) with the same affected frame rate;
- DMOS score was higher (worse) in the case of live streaming scenario (1 × 120 + 1 × 60 frame freeze) where frames were ‘lost’, compared with the case of stored video and 2 × 120 frame freezes; however, it was not clear by how much, as the former had a freeze at the end lasting for 60 frames;
- DMOS score was different for different video sequences and the same degree of AFR, which means that the final DMOS score depended also upon video content—a good example would be sequence number 11, with a very low DMOS score, as this sequence had low spatial and temporal information, so the long frame freeze did not have a higher impact on the subjective score.

It was assumed that the positions of all frame freezing events were known. This assumption was not unrealistic, as in a real transmission application it is possible to determine the starting time of the freezes from other parameters, such as headers in transport stream or in video compressed packets.

Furthermore, the proposed NR-FFM measure was evaluated, comparing its estimates and the database scores using data for frame freezing degradations only, as well as in combination with other quality measures, to obtain the overall correlation computed on all 200 distorted sequences.

### 3.2. VQEG HD5 Dataset

The VQEG HD5 dataset [28] is used later in this study to compare newly proposed objective measure NR-FFM and several existing objective measures designed for frame freezing degradation. Details of the LIVE mobile dataset have been previously discussed. The VQEG-HD5 dataset consists of several original full-HD video sequences, with 25 frames per second and 10 s overall duration. All video sequences can be downloaded from [29]. Degraded video sequences have general degradations due to the MPEG-2 (Moving Picture Experts Group) and H.264/AVC (advanced video coding) compression artefacts and packet losses, which introduce slicing errors and/or frame freezing; however, only in some video sequences do packet losses introduce frame freezing. Because of this, we used 7 video sequences (src01, src02, src04, src05, src06, src08, and src09) and 4 degradation types with frame freezing (hrc10, hrc11, hrc12, and hrc15), resulting overall in 28 degraded video sequences.

Specifically, degradations that were included in later comparison are, briefly:

- hrc10: H.264/AVC compression, bitrate 16 Mbit/s, 1-pass encoding, bursty packet loss, packet loss rate 0.125%; freezing errors;
- hrc11: H.264/AVC compression, bitrate 16 Mbit/s, 1-pass encoding, bursty packet loss, packet loss rate 0.25%; freezing errors;
- hrc12: H.264/AVC compression, bitrate 16 Mbit/s, 1-pass encoding, bursty packet loss, packet loss rate 0.5%; freezing errors;
- hrc15: H.264/AVC compression, bitrate 4 Mbit/s, 1-pass encoding, bursty packet loss, packet loss rate 0.25%; freezing errors.

## 4. NR-FFM Measure Development

To be able to further analyze video content, we used spatial and temporal activity indicators, the spatial information (SI), and the temporal information (TI) for all degraded video sequences. SI and TI are defined as follows [30]:

(1) $$\begin{array}{l} \mathrm{SI}=\max_{time}(std_{space}(Sobel_{H,V}(F_{n})))\\ \mathrm{TI}=\max_{time}(std_{space}(F_{n}-F_{n-1})) \end{array}$$

where *F_n_* represents luminance plane at time *n*, *Sobel* represents the Sobel operator and by convolvingwith 3 × 3 kernel, calculation of the SI is defined as maximal value of all Sobel-filtered frames standard deviation (*stdspace* ) values. By default, Sobel operator should be calculated for both horizontal and vertical edges. However, we later calculated SI for only horizontal (later described as SI_H_), only vertical (later described as SI_V_), and both horizontal and vertical edges (later described as SI_H,V_):

(2) $$\begin{array}{l} Sobel_{H}=\left[\begin{array}{ccc} -1 & -2 & -1\\ 0 & 0 & 0\\ 1 & 2 & 1 \end{array}\right];\;\\ Sobel_{V}=\left[\begin{array}{ccc} -1 & 0 & 1\\ -2 & 0 & 2\\ -1 & 0 & 1 \end{array}\right];\;\\ Sobel_{H,V}(F_{n})=\sqrt{Sobel_{H}^{2}(F_{n})+Sobel_{V}^{2}(F_{n})}. \end{array}$$

**Figure 3:**
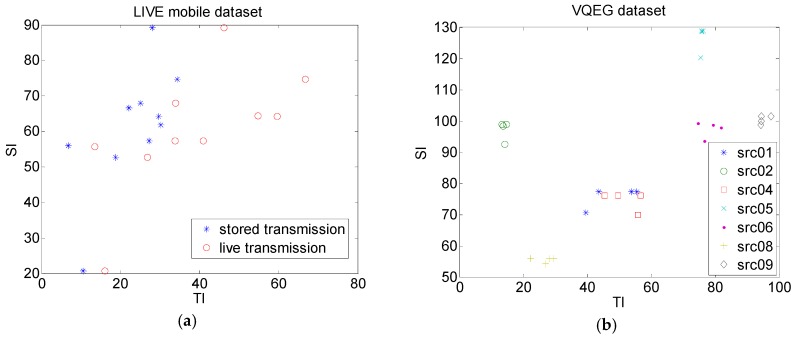
Temporal information versus spatial information (SI_H,V_) for: (**a**) LIVE mobile dataset, stored transmission scenario (blue) and live transmission scenario (red); (**b**) VQEG dataset (video sequences src01, src02, src04, src05, src06, src08, and src09 with degradations hrc10, hrc11, hrc12, and hrc15, previously described).

Results of TI versus SI are presented in Figure 3 for the LIVE mobile dataset and the VQEG dataset. For the LIVE mobile dataset, these were presented separately for the first three degradations (stored transmission) and fourth degradation (live transmission) separately. It can be seen that TI in the case of live transmission scenario was higher than in the case of the stored scenario. For the VQEG dataset, generally SI and TI characteristics were grouped for each video sequence. Also, it can be seen that the VQEG dataset was more diverse in terms of their dynamic characteristics when compared to the LIVE dataset. Later in this study, VQEG is used to compare proposed NR-FFM measure and several existing objective quality measures.

Firstly, we tried to find if there was a relationship between spatial information (SI), temporal information (TI), and DMOS scores, of all the 40 degraded sequences in the LIVE dataset. A genetic algorithm (GA) [31] was used to find a relationship between the four observed degradation types with the goal of maximizing Spearman’s correlation (GA population size was set to 50).

We then hypothesized the following form for the reference frame freezing measure (NR-FFM) for degraded video sequence j (j ∈ {1,40}):

(3) $$\text{NR-FFM}(j)=\left(\sum_{i=1}^{n}\mathrm{NFD}{\left(i,j\right)}^{\alpha }\right)\cdot \mathrm{SI}{\left(j\right)}^{\beta }\cdot \mathrm{TI}{\left(j\right)}^{\gamma }$$

where higher value means worse quality and *n* is number of freezes in one sequence *j*, NFD(*i*,*j*) is normalized frame duration of one freezing (normalized by sequence frame length), SI(*j*) and TI(*j*) are spatial and temporal information of the whole sequence *j* (according to the Equation (1)), respectively, and coefficients *α*, *β*, and *γ* were optimized using a GA to maximize Spearman’s correlation. Coefficient *α* takes into account impact of the duration of one frame freeze as well as overall frame freeze duration on final video quality. It should be expected that 0 < *α* < 1. *α* < 1 means that one freezing event with longer duration will have a better (lower) objective grade than few shorter freezing events (with same overall duration as one longer freezing event), which is in accordance with Figure 2. Also, *α* > 0 means that a longer frame freeze will have a higher impact (worse, higher objective grade) on video quality than a shorter frame freeze. Coefficients *β* and *γ* show the impact of spatial and temporal information on video quality. It would be expected that *β* and *γ* were higher than 0, meaning that higher spatial or temporal activity will have a higher influence on video quality.

## 5. Experiments and Results

### 5.1. Results Using Frame Freezing Degradations—Overall Measure, LIVE Mobile Dataset

We used LIVE mobile database frame freezing degradation sequences (40 video sequences) to train the proposed model from Equation (2). We calculated GA over the training set and tested calculated parameters *α* and *β* over the non-overlapping test set. We divided 10 original sequences in 7 or 8 for training (multiplied by 4 degradation levels per video sequence, giving 28 or 32 sequences overall) and 3 or 2 for testing (12 or 8 sequences overall). This gave (10 over 7) = 120 combinations or (10 over 8) = 45 combinations. Each combination was run 10 times and best (highest) Spearman’s correlation was taken as being the correct model. Afterwards, the model was tested on the remaining part of the frame-freezing dataset. The model was trained to have the highest possible Spearman’s correlation. Running GA 10 times (0 < *α* <1, *β* > 0, *γ* > 0, population size 50), using all combinations, we obtained the best results with mean *γ* near 0. Because of that, we decided to calculate NR-FFM using SI information only. By discarding TI from Equation (2), proposed NR-FFM should then have a following form:

(4) $$\text{NR-FFM}(j)=\left(\sum_{i=1}^{n}\mathrm{NFD}{\left(i,j\right)}^{\alpha }\right)\cdot \mathrm{SI}{\left(j\right)}^{\beta }.$$

In order to use them for comparison, Pearson’s, Spearman’s, and Kendall’s correlation were employed as follows. Pearson’s product–moment correlation coefficient was calculated as a normalized covariance between two variables, *x* and *y*:

(5) $$r_{xy}=\frac{\sum_{i=1}^{n}\left(x_{i}-\bar{x}\right)\cdot \left(y_{i}-\bar{y}\right)}{\left(n-1\right)\cdot s_{x}\cdot s_{y}}$$

where $x_{i}$ and $y_{i}$ are sample values (e.g., *x* is results from different objective measures and *y* is results from subjective tests), whereas $\bar{x}$ and $\bar{y}$ are sample mean and $s_{x}$ and $s_{y}$ are standard deviations from variables *x* and *y*. Spearman’s correlation assesses how well an arbitrary monotonic function can describe the relationship between two variables without making any assumptions about the frequency distribution of the variables [32]. It was calculated similarly as a Pearson’s correlation, Equation (5), but over ranked variables (every sample from both variables was firstly put in order: first, second, third, etc.; average rank was assigned to all tied ranks). Kendall’s correlation [33] is also a ranked correlation coefficient. It takes into account pair observations over ranked variables and calculates concordant pairs (sort order by variables *x* and by *y* having the same direction), discordant pairs (sort order by variables *x* and by *y* having the opposite direction), and possibly adjusts for tied pair observations (neither concordant nor discordant pairs). Generally, three types of Kendall’s correlation coefficient have been defined: $\tau_{a}$, $\tau_{b}$, and $\tau_{c}$. Kendall’s $\tau_{a}$ does not make any adjustment for ties, whereas $\tau_{b}$ and $\tau_{c}$ make adjustments. Kendall’s $\tau_{b}$ is used if the underlying scale of both variables has the same number of possible values (before ranking) and $\tau_{c}$ if they differ. As in our case, both variables *x* and *y* can have many different possible values, and Kendall’s $\tau_{b}$ coefficient (which is also defined in Matlab as the Kendall’s correlation coefficient,$\;\tau_{b}$) is used later in this study.

Results for Spearman’s and Pearson’s correlation are presented in Table 1, Table 2 and Table 3 for SI with only horizontal (SI_H_), both horizontal and vertical edges (SI_H,V_), and only vertical edges (SI_V_), respectively. Mean parameters α and β over non-overlapping test sets for those cases are presented in Table 4. After nonlinear regression using four parameter logistic functions Q_1_, Q_2,_ Q_3_, and Q_4_, Pearson’s correlation was calculated according to:

(6) $$\begin{array}{l} Q_{1}(z)=b_{1}\cdot (\frac{1}{2}-\frac{1}{1+e^{b_{2}\cdot (z-b_{3})}})+b_{4}\cdot z+b_{5}\\ Q_{2}(z)=\frac{b_{1}-b_{2}}{1+e^{\frac{z-b_{3}}{b_{4}}}}+b_{2}\\ Q_{3}(z)=b_{1}\cdot z^{3}+b_{2}\cdot z^{2}+b_{3}\cdot z+b_{4}\\ Q_{4}(z)=b_{1}\cdot z+b_{2} \end{array}.$$

Q_1_ and Q_2_ are defined in [34] and [35], respectively, whereas Q_3_ and Q_4_ represent cubic and linear fit.

From Table 1, Table 2 and Table 3, generally it can be concluded that the highest Pearson’s correlation was obtained using Q_1_ and Q_3_ fitting functions. Q_2_ had a somewhat lower correlation and Q_4_ function gave the lowest correlation (as could be expected from linear fitting function).

When the entire mobile dataset was used for training, we obtained Kendall’s, Spearman’s, and Pearson’s correlation according to Table 5. Pearson’s correlation was also calculated according to Equation (4) for Q_1_, Q_2_, Q_3_, and Q_4_.

When comparing Spearman’s and Pearson’s correlations from Table 1, Table 2 and Table 3, we obtained similar correlations in all three cases. However, according to Table 5, the best Kendall’s, Spearman’s, and Pearson’s correlations were obtained for SI with only a horizontal Sobel operator. Because of this, we further used this case (SI with horizontally calculated Sobel operator). Proposed NR-FFM should then have an explicit form:

(7) $$\text{NR-FFM}(j)=\left(\sum_{i=1}^{n}\mathrm{NFD}{\left(i,j\right)}^{0.6327}\right)\cdot \mathrm{SI}{\left(j\right)}^{0.1167}.$$

Table 6 presents Kendall’s, Spearman’s, and Pearson’s correlations using Equation (7) for LIVE mobile, stored transmission video sequences (30 video sequences), and LIVE mobile live transmission video sequences (10 video sequences) separately.

### 5.2. NR-FFM Measure: Comparison between “Mobile” and “Tablet” Sub-Dataset from LIVE Mobile Dataset

To further compare proposed NR-FFM measure, we also used DMOS tablet scores (20 video sequences with frame freezing degradations) with proposed NR-FFM from Equation (7). It has to be noted that these scores were obtained from the same video sequences as in the mobile dataset, however, they were shown on the tablet display.

**Figure 4:**
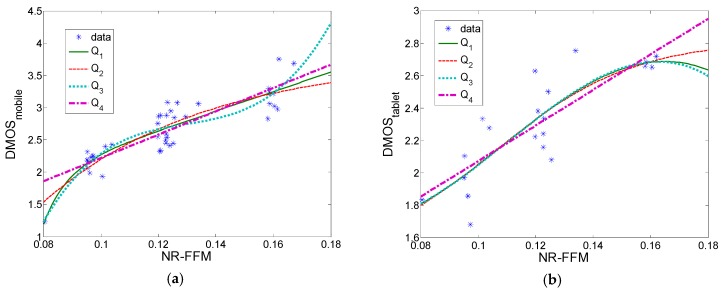
No-reference frame–freezing measure (NR-FFM) versus DMOS (trained using LIVE mobile sub-dataset): (**a**) LIVE mobile dataset; (**b**) LIVE tablet dataset.

If we used the NR-FFM measure defined in Equation (7), we obtained Kendall’s correlation of 0.6000; Spearman’s correlation of 0.8030; and Pearson’s correlation of 0.8357, 0.8353, 0.8358, and 0.8253 for Q_1_, Q_2,_ Q_3_, and Q_4_, respectively. Figure 4 shows the estimated values of NR-FFM measure versus the subjective DMOS scores using Equation (7). Also, Table 7 shows the fitting coefficients b_1_–b_5_, as defined in Equation (6), for this case.

Another solution to determine NR-FFM measure coefficients would be to train it on a tablet dataset (with 20 video sequences) and test it on a mobile dataset (with 40 video sequences). Training was performed equally as previously described for the overall mobile dataset. Parameters α and β in NR-FFM, Equation (4), were in this case determined to be 0.6356 and 0.1098, respectively. Table 8 presents Kendall’s, Spearman’s, and Pearson’s correlation (using Q_1_, Q_2_, Q_3_, and Q_4_ as fitting functions), also for this case.

### 5.3. Combined Results, Overall LIVE Mobile Dataset

To be able to combine the proposed NR measure with other objective measures, it has to be rescaled and to have the same regression as the targeted objective measure. One way of achieving this is by using the nonlinear regression model proposed in Equations (8) and (9), where free parameter δ was calculated using GA to give the highest possible Spearman’s correlation with DMOS. Rescaling was done using the maximum and minimum grade from the objective measure tested in advance for other degradation types, as well as from the proposed NR measure. We implemented proposed NR measure from Equation (4) on two reduced video quality measures, RVQM [12], and STRRED [10]. RVQM uses a scale of 0–1, where 0 means worst quality and 1 means no degradation. STRRED uses a scale from 0, where 0 means no degradation.

RVQM measure is based on 3D (three-dimensional) steerable wavelet transform (Riesz transform [36]) and modified SSIM (structural similarity index [6]) measure (using only contrast and structure terms). Here, we resized video frames into rectangular cuboid with size 64 × 64 per frame with 32 frames, and averaging filter with four pixels. Step was set to half the size of the tested cube in time, with 16 pixels. Modified SSIM index was calculated from the third component from the first Riesz order decomposition with Shannon prefiltering and Simoncelli filter, as it was concluded in [12].

STRRED (spatio-temporal reduced reference entropic differences) is another measure with variable range of the reference information (from single scalar to the full reference information). It combines spatial RRED index [37] (SRRED) and temporal RRED index (TRRED).

In the case of implementing the proposed measure of frame freezing degradations in RVQM, rescaling was done according to

(8) $${\text{NR-FFM}}_{\mathrm{RVQM}}=1-\frac{1-min(RVQM)}{max({\text{NR-FFM}}^{\delta_{1}})}\cdot {\text{NR-FFM}}^{\delta_{1}},$$

and in the case of STRRED according to Equation (8):

(9) $${\text{NR-FFM}}_{STRRED}=\frac{\max(\mathrm{STRRED})}{\max({\text{NR-FFM}}^{\delta_{2}})}\cdot {\text{NR-FFM}}^{\delta_{2}}$$

At the end, combined measures could be calculated as follows:

(10) $$\begin{array}{l} RVQM_{combined}={\text{NR-FFM}}_{RVQM}\cdot RVQM\\ STRRED_{combined}=({\text{NR-FFM}}_{STRRED}+1)\cdot (STRRED+1) \end{array}.$$

In Equation (10), STRRED combined was rescaled to start from 1 (meaning perfect quality), otherwise the combined measure would give 0 even if only one part of the measure gave a perfect grade (zero).

As in the previous case (only frame-freezing degradations), we divided the dataset in seven or eight (original video sequences) for training and the rest for testing (three and two, respectively). This gave overall (multiplied by four degradation levels per degradation type and five degradation types) 140 or 160 video sequences for training. The rest of the non-overlapping video sequences were used for testing (60 and 40, respectively). Results for Kendall’s, Spearman’s, and Pearson’s correlation between RVQM, STRRED, and DMOS are presented in Table 9.

If the entire dataset was used for training in Equations (8) and (9), δ was calculated using GA running 10 times (again with target to maximize Spearman’s correlation, with α = 0.6327 and β = 0.1167, from Equation (7)); δ1 = 3.2023 and δ2 = 3.8115. In this case, rescaled measures can be explicitly written as in following equations:

(11) $${\text{NR-FFM}}_{RVQM}=1-64.6136\cdot {\text{NR-FFM}}^{3.2023},$$

(12) $${\text{NR-FFM}}_{STRRED}=3.3464\cdot 10^{5}\cdot {\text{NR-FFM}}^{3.8115}.$$

**Figure 5:**
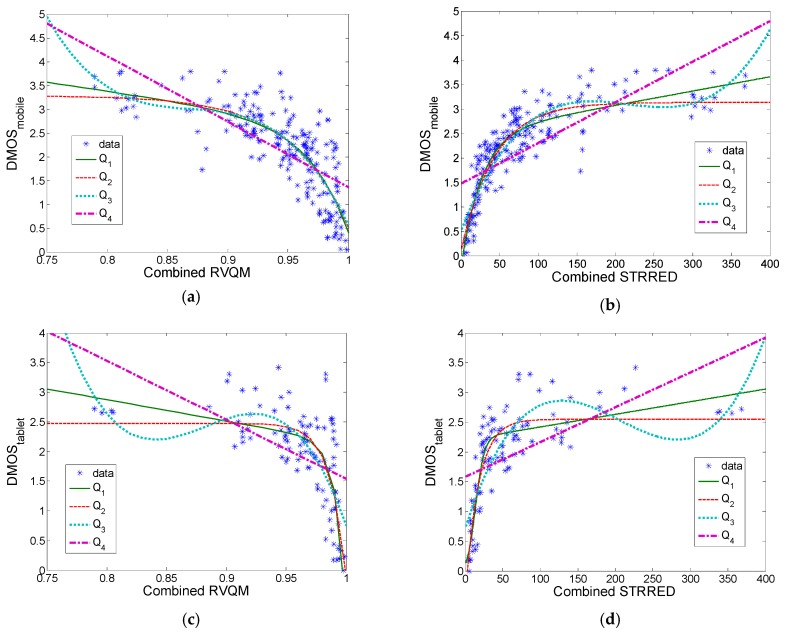
Combined objective measure: (**a**) RVQM (reduced video quality measure), mobile dataset; (**b**) STRRED (spatio-temporal reduced reference entropic differences), mobile dataset; (**c**) RVQM, tablet dataset; (**d**) STRRED, tablet dataset.

Figure 5 shows the relationship between combined RVQM measure and combined STRRED measure with DMOS scores (200 degraded video sequences for mobile dataset and 100 degraded sequences for tablet dataset), using Equations (10)–(12). In the tablet dataset, the same coefficients have been used as in the mobile dataset, Equations (11) and (12).

Table 10 shows Kendall’s, Spearman’s, and Pearson’s correlation, before and after combining RVQM and STRRED measures with NR-FFM measure, using the entire mobile dataset. Table 11 shows Kendall’s, Spearman’s, and Pearson’s correlation for the tablet dataset using the fitting coefficient from the mobile dataset study, Equations (11) and (12).

Pearson’s correlation was calculated after nonlinear regression using Equation (6). In these tables, results for VQM measure [13] that incorporates variable frame delay distortion and full or reduced reference calibration, called VQM-VFD-FR or VQM-VFD-RR measures, have also been included from [17]. It can be concluded that NR-FFM can be included in other measures without lowering overall correlation with DMOS. Regarding the fitting functions for Pearson’s correlation, Q_1_, Q_2_, and Q_3_ have similar correlations, whereas Q_4_ produces a much lower correlation. This means that the linear fitting function Q_4_ cannot be used in this case. It has to be also noted that in this database, only one part from Equation (10) has an influence on the final objective metric (frame-freezing degradation or any other degradation type), whereas the other part shows perfect quality (with score 1).

### 5.4. NR-FFM Measure Comparison with Other Objective Measures for Frame Freezing Degradations 

In this subsection we will compare newly proposed NR-FFM measure with several existing measures, named “Borer” (no reference objective measure) [19], “FDF” (no reference objective measure) [20], “FDF-RR” (reduced reference objective measure) [20], and “Quanhuyn” (no reference objective measure) [15]. Table 12 presents Kendall’s, Spearman’s, and Pearson’s correlation for the LIVE mobile dataset (40 degraded video sequences described earlier), whereas Table 13 presents Kendall’s, Spearman’s, and Pearson’s correlation for the VQEG dataset (28 degraded video sequences described earlier). In both tables, Borer measure was calculated according to the paper [19], but with the motion intensity value set to 1 for all degraded video sequences (otherwise, correlation was always lower). FDF and FDF-RR measures were calculated using Matlab code from [38] (with read_avi function from command line video quality metric, CVQM, MATLAB source code for CVQM version 3.0 [39]). Quanhuyn measure was also calculated according to the paper [15]. Our proposed measure, NR-FFM, was in Table 12 equal to that in Table 1, training-test ratio 8:2, mean value. In Table 13, NR-FFM was calculated according to Equation (7) and using SI_H_ (from Equations (1) and (2)) as spatial information values.

## 6. Discussion

In this paper we developed no reference objective measure NR-FFM and tested it using two different datasets, LIVE mobile and VQEG-HD5. NR-FFM measure was also compared with some existing objective measures for frame freezing degradations named Borer (no-reference), FDF (no-reference), Quanhuyn (no-reference), and FDF-RR (reduced reference). In the LIVE mobile dataset, NR-FFM gave the best correlation results between all tested measures (Table 12). In the VQEG-HD5 dataset, NR-FFM (which was trained on the LIVE mobile dataset) had the best Pearson’s correlation for Q1, Q2, and Q3 fitting functions (Table 13). Borer and FDF-RR measures obtained somewhat higher Spearman’s and Kendall’s correlations (Table 13).

The main difference between LIVE mobile frame freezing degradations and VQEG-HD5 frame freezing degradations were the type and overall duration of the freezing occurrences. In the LIVE mobile dataset, there were online degradations (due to, for example, packet losses), which resulted in frame drops and offline degradations (due to, for example, packet delay), where no frame was actually lost, only delayed. Also, in the LIVE mobile dataset, overall freezing duration was generally the same—in offline frame freezing 8 × 1 s long, 4 × 2 s long, and 2 × 4 s long. Only online freezing had one 4 s long freeze and one 2 s long freeze. In the VQEG-HD5 dataset, all freezing frames were online degradations, resulting in frame drops. Also, higher packet loss ratio (PLR) resulted in longer frame freezes and, consequently, more lost frames. This meant that overall frame freezing duration was different for different degradation types (in the VQEG-HD5 dataset this was due to the different PLR ratio). Probably because of this, the NR measures FDF and Borer had lower correlation in the LIVE mobile dataset compared to the VQEG-HD5 dataset. Also, motion intensity or temporal information (or any other measure that would take differences between consecutive frames into account) did not have a higher value in offline frame freezing compared to the original video sequence (and that degradation was present only in the LIVE mobile dataset). NR-FFM measure had the best correlation in the LIVE mobile dataset compared to the other tested measures, probably also because it was calculated only on the basis of frame freezing duration and spatial information. NR-FFM measure also had different values for the equal overall frame freezing, but with different numbers of occurrences (see Equation (7)). Nonetheless, in the VQEG-HD5 dataset, NR-FFM had similar correlation to Borer and FDF measures without pretrained values on the VQEG-HD5 dataset.

When comparing online degradation (e.g., stored video sequences) and offline degradation (e.g., live video sequences) types in the LIVE mobile dataset (Table 6), offline degradation had higher correlation. However, in online degradation there were only 10 video sequences with equal degradation type—two equally spaced frame drops that were 4 and 2 s long (3–7 and 13–15 s in each video sequence); thus, these correlation coefficients had lower confidence. Also, compared with Table 13, also with only an online degradation type, NR-FFM had in this case a higher correlation for the tested VQEG-HD5 dataset, probably due to the different degradation levels for each video sequence (4 video sequences), as well as more tested video sequences (28 video sequences overall).

NR-FFM measure was also tested using both the LIVE mobile (40 video sequences) and LIVE tablet study (20 video sequences) and was trained/tested using another dataset, showing similar fitting coefficients and correlations in both cases (trained on the LIVE mobile and tested on the LIVE tablet and vice versa).

Furthermore, we combined the proposed NR-FFM measure with some existing reduced-reference measures (RVQM and STRRED) and tested it using all video sequences from the LIVE mobile/tablet dataset. Results have shown that it is possible to obtain similar correlation for the overall combined objective measure using all five degradation types in the LIVE mobile dataset (Table 10 and Table 11).

## 7. Conclusions

In this paper we proposed a new NR-FFM measure for frame freezing degradations. It can be used only for this degradation type or in combination with other degradation types, not lowering their overall correlation (which we checked on RVQM and STRRED video quality measures in the LIVE mobile and tablet video databases).

Future research may be based on temporal information (or motion intensity) implementation in the proposed NR-FFM measure to obtain higher correlation with subjective grades for video sequences with both offline and online frame freezing. Alternatively, different formulae maybe defined for spatial and temporal information instead of the proposed formula in Equation (7). A larger dataset should also then be developed to be able to calculate correlations with higher confidence.

## Figures and Tables

**Table 1:** Kendall’s, Spearman’s, and Pearson’s correlation for different train-test dataset ratio—SI_H_.

Train-Test Ratio	Kendall’s Correlation		Spearman’s Correlation		Pearson’s Correlation, Q_1_		Pearson’s Correlation, Q_2_		Pearson’s Correlation, Q_3_		Pearson’s Correlation, Q_4_	
	Mean	Median	Mean	Median	Mean	Median	Mean	Median	Mean	Median	Mean	Median
7–3	0.6309	0.6364	0.7878	0.8042	0.8761	0.8809	0.8542	0.8619	0.8773	0.8838	0.8294	0.8381
8–2	0.6592	0.6429	0.7960	0.8095	0.9090	0.9018	0.8903	0.8820	0.9110	0.9133	0.8454	0.8651

**Table 2:** Kendall’s, Spearman’s, and Pearson’s correlation for different train-test dataset ratio—SI_H,V_.

Train-Test Ratio	Kendall’s Correlation		Spearman’s Correlation		Pearson’s Correlation, Q_1_		Pearson’s Correlation, Q_2_		Pearson’s Correlation, Q_3_		Pearson’s Correlation, Q_4_	
	Mean	Median	Mean	Median	Mean	Median	Mean	Median	Mean	Median	Mean	Median
7–3	0.6433	0.6364	0.7964	0.7980	0.8768	0.8808	0.8620	0.8603	0.8811	0.8834	0.8328	0.8422
8–2	0.6796	0.6429	0.8101	0.8095	0.9008	0.8983	0.8882	0.8894	0.9109	0.9203	0.8608	0.8680

**Table 3:** Kendall’s, Spearman’s, and Pearson’s correlation for different train-test dataset ratio—SI_V_.

Train-Test Ratio	Kendall’s Correlation		Spearman’s Correlation		Pearson’s Correlation, Q_1_		Pearson’s Correlation, Q_2_		Pearson’s Correlation, Q_3_		Pearson’s Correlation, Q_4_	
	Mean	Median	Mean	Median	Mean	Median	Mean	Median	Mean	Median	Mean	Median
7–3	0.6536	0.6667	0.8089	0.8112	0.8887	0.8914	0.8705	0.8710	0.8882	0.8930	0.8396	0.8447
8–2	0.6869	0.6429	0.8144	0.8095	0.9095	0.9078	0.8927	0.8884	0.9098	0.9099	0.8550	0.8620

**Table 4:** Mean parameters α and β over non-overlapping test sets. SI_H_: only horizontal spatial information, SI_H,V_: horizontal and vertical spatial information, SI_V_: only vertical spatial information.

Train-Test Ratio	SI_H_		SI_H,V_		SI_V_	
	Mean α	Mean β	Mean α	Mean β	Mean α	Mean β
7–3	0.5531	0.1769	0.4962	0.1211	0.4727	0.1308
8–2	0.5861	0.1550	0.5407	0.0962	0.4721	0.1446

**Table 5:** Kendall’s, Spearman’s, and Pearson’s correlation for the overall mobile dataset, different SI calculations (best values are marked in bold). H: horizontal, V: vertical.

SI Type	Kendall’s Correlation	Spearman’s Correlation	Pearson’s Correlation Using Q_1_	Pearson’s Correlation Using Q_2_	Pearson’s Correlation Using Q_3_	Pearson’s Correlation Using Q_4_	α	β
H only	**0.7026**	**0.8842**	**0.8952**	**0.8840**	**0.8978**	**0.8868**	0.6327	0.1167
H and V	0.6795	0.8598	0.8919	0.8790	0.8936	0.8574	0.5824	0.1672
V only	0.6846	0.8683	0.8882	0.8677	0.8913	0.8353	0.2917	0.2127

**Table 6:** Kendall’s, Spearman’s, and Pearson’s correlation, using LIVE mobile, stored transmission video sequences, and LIVE mobile live transmission video sequences.

LIVE Dataset	Kendall’s Correlation	Spearman’s Correlation	Pearson’s Correlation Using Q_1_	Pearson’s Correlation Using Q_2_	Pearson’s Correlation Using Q_3_	Pearson’s Correlation Using Q_4_
Stored transmission	0.7103	0.8919	0.9181	0.9084	0.9226	0.8906
Live transmission	0.3333	0.3576	0.6526	0.6150	0.7008	0.4334

**Table 7:** Fitting coefficients b_1_–b_5_.

Fitted Function	Mobile Dataset					Tablet Dataset				
	b_1_	b_2_	b_3_	b_4_	b_5_	b_1_	b_2_	b_3_	b_4_	b_5_
Q_1_	84	123.8	0.04294	15.1	−41.17	18.72	16.42	0.1091	−62.74	9.021
Q_2_	−500.9	3.758	−0.2237	0.05604	-	1.442	2.818	0.1054	0.02429	-
Q_3_	9429	−3667	482.7	−18.74	-	−1687	555.6	−46.82	2.863	-
Q_4_	18.07	0.4138	-	-	-	10.98	0.9761	-	-	-

**Table 8:** Kendall’s, Spearman’s, and Pearson’s correlation for the overall frame freezing dataset, using the LIVE tablet sub-dataset for training and the LIVE mobile sub-dataset for testing.

LIVE Dataset	Kendall’s Correlation	Spearman’s Correlation	Pearson’s Correlation Using Q_1_	Pearson’s Correlation Using Q_2_	Pearson’s Correlation Using Q_3_	Pearson’s Correlation Using Q_4_
Tablet	0.6000	0.8030	0.8422	0.8417	0.8422	0.8312
Mobile	0.7026	0.8842	0.8957	0.8838	0.8978	0.8668

**Table 9:** Kendall’s, Spearman’s, and Pearson’s correlation for different train-test dataset ratio (best values are marked in bold).

Measure	Train-Test Ratio	Kendall’s Correlation		Spearman’s Correlation		Pearson’s Correlation, Q_1_		Pearson’s Correlation, Q_2_		Pearson’s Correlation, Q_3_		Pearson’s Correlation, Q_4_	
		Mean	Median	Mean	Median	Mean	Median	Mean	Median	Mean	Median	Mean	Median
RVQM	7–3	0.6255	0.6193	0.8065	0.7995	0.8390	0.8383	0.8290	0.8262	0.8272	0.8281	0.7019	0.7052
	8–2	0.6494	0.6692	0.8210	0.8396	0.8669	0.8859	0.8527	0.8800	0.8494	0.8804	0.7012	**0.7120**
STRRED	7–3	0.7115	0.7173	0.8773	0.8852	0.9131	0.9166	0.9059	0.9122	0.8895	0.8918	**0.7049**	0.7083
	8–2	**0.7204**	**0.7283**	**0.8785**	**0.8861**	**0.9219**	**0.9256**	**0.9150**	**0.9214**	**0.8982**	**0.9044**	0.7004	0.7043

**Table 10:** Kendall’s, Spearman’s, and Pearson’s correlation, before and after combining RVQM and STRRED with freezing degradations; VQM-VFD-RR and VQM-VFD-FR correlation from [17]; mobile dataset (best values are marked in bold).

Measure	Kendall’s Correlation		Spearman’s Correlation		Pearson’s Correlation Using Q_1_		Pearson’s Correlation Using Q_2_		Pearson’s Correlation Using Q_3_		Pearson’s Correlation Using Q_4_	
	Before Merging (160 Videos)	After Merging (200 Videos)	Before Merging (160 Videos)	After Merging (200 Videos)	Before Merging (160 Videos)	After Merging (200 Videos)	Before Merging (160 Videos)	After Merging (200 Videos)	Before Merging (160 Videos)	After Merging (200 Videos)	Before Merging (160 Videos)	After Merging (200 Videos)
RVQM	0.5779	0.6129	0.7697	0.8034	0.7790	0.8045	0.7780	0.8032	0.7780	0.8039	0.7108	0.7142
STRRED	**0.7214**	**0.7237**	**0.8902**	**0.8929**	**0.9125**	**0.9156**	**0.9062**	**0.9085**	**0.8921**	**0.8936**	**0.7542**	**0.7275**
VQM-VFD-RR	-	-	-	0.8301	-	0.8645	-	-	-	-	-	-
VQM-VFD-FR	-	-	-	0.8295	-	0.8631	-	-	-	-	-	-

**Table 11:** Kendall’s, Spearman’s, and Pearson’s correlation, before and after combining RVQM and STRRED with freezing degradations; VQM-VFD-RR and VQM-VFD-FR correlation from [17]; tablet dataset (best values are marked in bold).

Measure	Kendall’s Correlation		Spearman’s Correlation		Pearson’s Correlation Using Q_1_		Pearson’s Correlation Using Q_2_		Pearson’s Correlation Using Q_3_		Pearson’s Correlation Using Q_4_	
	Before Merging (80 Videos)	After Merging (100 Videos)	Before Merging (80 Videos)	After Merging (100 Videos)	Before Merging (80 Videos)	After Merging (100 Videos)	Before Merging (80 Videos)	After Merging (100 Videos)	Before Merging (80 Videos)	After Merging (100 Videos)	Before Merging (80 Videos)	After Merging (100 Videos)
RVQM	0.5128	0.4972	0.6704	0.6621	0.7236	0.7362	0.7192	0.7261	0.7244	0.6991	0.6621	0.5206
STRRED	**0.6287**	**0.5853**	**0.8104**	0.7731	**0.8844**	**0.8645**	**0.8648**	**0.8563**	**0.8418**	**0.7848**	**0.6814**	**0.5433**
VQM-VFD-RR	-	-	-	0.8133	-	0.8110	-	-	-	-	-	-
VQM-VFD-FR	-	-	-	**0.8385**	-	0.8347	-	-	-	-	-	-

**Table 12:** Kendall’s, Spearman’s, and Pearson’s correlation for the LIVE mobile dataset—40 degraded video sequences (best values are marked in bold). Borer: no reference objective measure, FDF: no reference objective measure, FDF-RR: reduced reference objective measure, Quanhuyn: no reference objective measure.

Measure	Kendall’s Correlation	Spearman’s Correlation	Pearson’s Correlation Using Q_1_	Pearson’s Correlation Using Q_2_	Pearson’s Correlation Using Q_3_	Pearson’s Correlation Using Q_4_
NR-FFM_8:2	0.6592	0.7960	0.9090	0.8903	0.9110	0.8454
Borer	0.3663	0.5251	0.8286	0.7216	0.8232	0.0896
FDF	0.0725	0.0873	0.2590	0.2553	0.2590	0.2064
FDF-RR	0.0290	0.0375	0.2590	0.1572	0.2590	0.0790
Quanhuyn	0.3203	0.4594	0.7443	0.6221	0.7443	0.4253

**Table 13:** Kendall’s, Spearman’s, and Pearson’s correlation for the VQEG dataset—28 degraded video sequences (best values are marked in bold).

Measure	Kendall’s Correlation	Spearman’s Correlation	Pearson’s Correlation Using Q_1_	Pearson’s Correlation Using Q_2_	Pearson’s Correlation Using Q_3_	Pearson’s Correlation Using Q_4_
NR-FFM	0.6596	0.8523	0.8845	0.8736	0.8750	0.8425
Borer	0.6916	0.8685	0.8736	0.8729	0.8735	0.8496
FDF	0.6344	0.8240	0.8206	0.8187	0.8198	0.8072
FDF-RR	0.6756	0.8576	0.8527	0.8534	0.8529	0.8372
Quanhuyn	0.4147	0.5129	0.6056	0.5592	0.5900	0.4468
